# Alzheimer Disease: Controversies in Basic Science Research, Different Theories, and Reasons for Failed Trials

**DOI:** 10.3390/biomedicines9030254

**Published:** 2021-03-05

**Authors:** Farid Rahimi

**Affiliations:** Division of Biomedical Science and Biochemistry, Research School of Biology, ANU College of Science, The Australian National University, Canberra, ACT 2600, Australia; farid.rahimi@anu.edu.au

Dementia comprises a collection of cognitive and sensory symptoms, including memory loss, communication difficulties, difficulty in planning and problem solving, disorientation and confusion, compromised olfaction, loss of visual perception, agnosia; and psychological symptoms, including personality and behavioral changes, depression, anxiety, hallucination, mood swings, agitation, and apathy. Dementia can be caused by a spectrum of different brain afflictions. The most common cause of dementia is Alzheimer disease (AD) or senile dementia of Alzheimer type. Other types include vascular dementia, dementia with Lewy bodies, frontotemporal dementia, alcohol-related dementia, Down syndrome, Parkinson disease dementia, and HIV-associated dementia [1,2].

AD, which accounts for 60–80% of diagnosed dementia cases, is a predominant, devastating, and chronic disease that begins with episodic memory lapses (amnesia) and progresses to mood swings, personality changes, impaired communication (aphasia), and deficits in voluntary motor skills (apraxia), culminating in decline of mental capacities (agnosia), and eventually and sadly death. AD typically afflicts patients in their eighth or ninth decade of life, with its incidence rising after 65 years of age. The global statistics of AD prevalence are ominous [3], and the psychological and financial burdens of care on dementia families and on global healthcare systems are immeasurable. The forbidding statistics have established AD as a profoundly alarming global health crisis.

Despite the grim statistics, research into AD has progressed steadily and strongly, generating a vast collection of complex literature since the original description of AD in 1901 by Alois Alzheimer [4]. The expansive, and sometimes confusing, AD literature requires selective, critical, and in-depth scrutiny of the AD facts before designing, undertaking, or publishing purposeful, worthwhile, informative, unbiased, and pioneering studies. Different, and sometimes, conflicting theories about AD etiology abound, while multiple clinical trials based on predominant theories of AD etiology have so far failed to produce effective disease-modifying or curative treatments [5]. Extant, approved AD drugs provide only modest symptomatic relief.

In this Special Issue in *Biomedicines*, I hoped to gather discussions and recent understandings of the multifactorial nature of AD etiology, while encouraging authors, editors, and the funding bodies to consider alternative aspects of this debilitating and emotionally and financially costly disease that has no approved curative treatment.

In “Exposure to CuO Nanoparticles Mediates NFκB Activation and Enhances Amyloid Precursor Protein Expression”, Mou et al. used a cellular model to show that exposure to CuO nanoparticles increases NFκB expression, which, in turn, augments expression of the amyloid β-protein precursor protein (APP) [6]. Redox, NFκB activation, and neuroinflammation were shown again to be tied to APP expression [7,8]. Mou and colleagues also confirmed that when p65 was inhibited by siRNA, cell stimulation by TNFα or CuO nanoparticles did not cause APP production [6].

Dhakal and Macreadie, in their manuscript titled “Tyramine and Amyloid Beta 42: A Toxic Synergy”, assessed the role of trace amine, tyramine, in control yeast, and yeast producing Aβ42 or GFP-tagged Aβ42 [9]. The authors reported that tyramine treatment of control yeast cells increased production of the reactive oxygen species, whereas cells expressing Aβ42 or GFP–Aβ42 produced significantly higher levels of the reactive oxygen species, suggesting a cooperative toxic effect between tyramine and Aβ42 [9]. Tyramine was also reported to inhibit respiratory growth of the yeast and damage the mitochondrial DNA in the presence of Aβ42 [9].

Ataur Rahman et al. provided a review titled “Modulatory Effects of Autophagy on APP Processing as a Potential Treatment Target for Alzheimer’s Disease”, highlighting the contemporary findings regarding the regulation of autophagy and APP processing in AD, while summarizing the use of some small molecules and natural compounds that modulate autophagy to facilitate APP and Aβ clearance [10]. The authors detailed the background of the mTOR-dependent and mTOR-independent autophagy pathways, the roles of APP in neurons, enzymatic processing of APP in AD, APP and Aβ processing by autophagy pathways, and the consequences of dysfunctional autophagy pathways on Aβ processing [10].

Considering the failures of the antibody trials against Aβ and tau, in the article titled “Novel MRI Techniques Identifying Vascular Leak and Paravascular Flow Reduction in Early Alzheimer Disease”, Charles R. Joseph introduced his review article by providing the background on the regulation of fluids and metabolites in the brain parenchyma by the blood–brain barrier, the glymphatic system, and the microglial system; then, the author concentrated on the breakdown of the blood–brain barrier during early AD progression, exploring the relationship between the blood–brain barrier, the glymphatic system, and the microglial surveillance system [11]. Highlighting the importance of minimally invasive procedures to examine the integrity of the blood–brain barrier and the glymphatic system and correlating them with clinical outcomes, the author then discussed the strengths and weaknesses of the following imaging techniques: high-resolution, dynamic contrast-enhanced magnetic resonance imaging (MRI); arterial spin-labelling MRI; arterial spin-labelling perfusion MRI; and 3-dimensional pulsed arterial spin-labelling MRI [11]. The author concludes that high-resolution, dynamic contrast-enhanced MRI could demonstrate the blood–barrier leakage, and the 3-dimensional pulsed arterial spin-labelling MRI could detect significant delays in glymphatic clearance in patients with AD [11].

In the review article titled “Reasons for Failed Trials of Disease-Modifying Treatments for Alzheimer Disease and Their Contribution in Recent Research”, Yiannopoulou et al. explained why clinical trials have yielded no new approved drug for AD treatment since 2003. The authors first summarized the basic pathology of AD and described why the candidate disease-modifying therapeutics of AD had failed so far. Examples included γ-secretase inhibitors, BACE1 inhibitors, monoclonal antibodies directed against fibrillar and soluble Aβ assemblies, and an inhibitor of tau aggregation [12]. The authors then discussed and summarized many novel biomarkers that were relevant to the underlying pathological mechanisms of AD; these included biomarkers relevant to Aβ metabolism and aggregation, inflammation, glial activation, vasculopathy, synaptic dysfunction, α-synuclein pathology, TDP-43, iron metabolism, oxidative stress, and neuronal biomarkers [12].

I thank all the authors, peer-reviewers, the editors, and the *Biomedicines* team for their contributions to this Special Issue.
